# Co-option of the cardiac transcription factor *Nkx2.5* during development of the emu wing

**DOI:** 10.1038/s41467-017-00112-7

**Published:** 2017-07-25

**Authors:** Peter G. Farlie, Nadia M. Davidson, Naomi L. Baker, Mai Raabus, Kelly N. Roeszler, Claire Hirst, Andrew Major, Mylene M. Mariette, David M. Lambert, Alicia Oshlack, Craig A. Smith

**Affiliations:** 1 Murdoch Children’s Research Institute, Royal Children’s Hospital, Parkville, VIC 3052 Australia; 2 Department of Paediatrics, University of Melbourne, Parkville, VIC 3052 Australia; 3 Biomedicine Discovery Institute, Department of Anatomy and Developmental Biology, Monash University, Clayton, VIC 3800 Australia; 4 Centre for Integrative Ecology, School of Life and Environmental Sciences, Deakin University, Waurn Ponds, VIC 3216 Australia; 5 Environmental Futures Research Institute, Griffith University, Nathan, QLD 4111 Australia

## Abstract

The ratites are a distinctive clade of flightless birds, typified by the emu and ostrich that have acquired a range of unique anatomical characteristics since diverging from basal Aves at least 100 million years ago. The emu possesses a vestigial wing with a single digit and greatly reduced forelimb musculature. However, the embryological basis of wing reduction and other anatomical changes associated with loss of flight are unclear. Here we report a previously unknown co-option of the cardiac transcription factor *Nkx2.5* to the forelimb in the emu embryo, but not in ostrich, or chicken and zebra finch, which have fully developed wings. *Nkx2.5* is expressed in emu limb bud mesenchyme and maturing wing muscle, and mis-expression of *Nkx2.5* throughout the limb bud in chick results in wing reductions. We propose that *Nkx2.5* functions to inhibit early limb bud expansion and later muscle growth during development of the vestigial emu wing.

## Abstract (web summary)

The transcription factor Nkx2.5 is essential for heart development. Here, the authors identify a previously unknown expression domain for Nkx2.5 in the emu wing and explore its role in diminished wing bud development in the flightless emu, compared with three other birds that have functional wings.

## Introduction

The Paleognathes are a distinctive group of birds represented by the volant Tinamou and seven genera of flightless ratites that diverged from basal Aves over 100 million years ago^1^. Morphological phylogenetic analyses initially suggested a monophyletic origin of the ratites with tinamou as the sister taxa, implying radiation from a common flightless ancestor^2, 3^. However, more recent genetic data indicating closest relationships between species separated geographically through deep evolutionary time have prompted the proposal of polyphyly and flightlessness as a convergent trait within the ratites^4, 5^. While these phylogenetic analyses infer evolutionary relationships, they do not address the embryonic origins of anatomical alterations characterizing ratite evolution. The developmental strategies through which individual ratites lost flight are currently unknown.

**Fig. 1:**
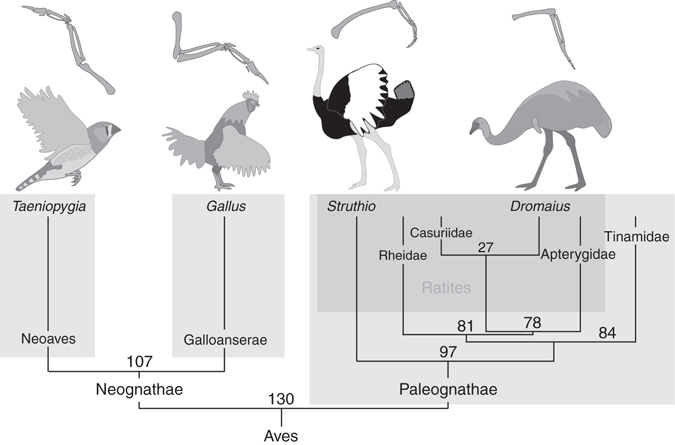
Wing morphology and evolutionary relationships within Aves. *Diagram* illustrating the morphological variation in wing structure and skeletal configuration between the four avian species investigated, and *chronogram* illustrating the evolutionary relationships and times of divergence within Aves. *Numbers* above nodes represent time in millions of years before present. *Shaded areas* indicate each of the three major subdivisions within Aves. Family tree constructed from data in refs. ^1, 4^

Subtle changes in the expression of known limb-patterning genes have been shown to underlie structural variation among diverse animal groups^6–9^. During embryonic development, the morphology of the vertebrate limb is determined during early limb bud outgrowth when patterning genes establish the basic configuration of skeletal elements including digit number^10^. The chicken and other volant (flying) birds such as the zebra finch have functional wings, characterized by well-developed stylopod (humerus) and zeugopod (radius and ulna) and an autopod (carpals, metacarpals, and phalanges) with three digits (Fig. 1). In contrast, wing morphology among the flightless ratites varies significantly from flighted Aves. The mature wing of the emu is vestigial, with no known function, and exhibits a substantially reduced and morphologically highly variable wing with an autopod harboring a single digit^11^. In contrast, the ostrich and rhea possess functional wings with strongly reduced zeugopod and autopod harboring three digits^12, 13^. The ratites have long been viewed as monophyletic^2, 3, 14^. However, recent phylogenetic analysis strongly supports a polyphyletic origin for ratites, indicating that each of the ratite genera lost flight independently^4, 5^. Developmental studies have an important role in providing mechanistic data to support hypotheses on phylogeny. In this instance, a monophyletic model of flightlessness within the ratites would suggest a common embryological mechanism resulting in wing reduction, while a polyphyletic origin of would be supported by multiple mechanisms.

Our understanding of the variation in the limb growth and patterning mechanisms resulting in wing reductions in the emu and other birds is rudimentary. An essential initiator of forelimb development is the transcription factor *Tbx5*. Loss of *Tbx5* in mice results in the complete absence of forelimb development, but normal hindlimb development and mutation of *TBX5* in humans result in Holt-Oram syndrome characterized by upper limb and heart defects^15–17^. Given its conservation and importance to forelimb bud initiation, alterations to *Tbx5* expression would be a potential mechanism underlying vestigial development of the emu wing. It has previously been reported that there is a delay in the initiation of *Tbx5* expression during the stages preceding limb bud outgrowth in the emu^18^. Similarly, our previous work has revealed alterations in the expression of a number of patterning factors such as *Sonic Hedgehog* and *Patched1*, in embryonic emu limb buds^19^. These altered expression patterns may simply be the result of delayed development and reduced size of the emu forelimb bud, or they could be causative. However, the functional consequences of such delayed or reduced expression in emu wing development have not been examined.

Through transcriptional profiling of embryonic emu wing buds, we have identified a novel domain of *Nkx2.5* expression within the developing emu wing. Intriguingly, this mRNA is not present in ostrich, chick, and zebra finch embryos. *Nkx2.5* has not previously been associated with limb patterning or development in any species. Early expression is detected throughout the nascent limb bud mesenchyme and extends rostrally in a superficial domain connecting to the 6th branchial artery. As development proceeds, expression becomes progressively restricted to superficial domains within the limb bud corresponding to myogenic precursors and expression is sustained in maturing muscle at fetal stages. Emu wing muscle is dramatically reduced in size and exhibits reduced muscle fiber diameter relative to leg muscle. Functional studies performed in the chicken embryo reveal that mis-expression of *Nkx2.5* in the myogenic lineage does not alter wing morphology, while a global mis-expression in the limb bud results in reduced wing outgrowth and morphological changes including digit loss. Surprisingly, analysis of gene expression profiles in morphologically affected limb buds following *Nkx2.5* mis-expression reveals no changes in established limb-patterning genes. However, *Nkx2.5* mis-expression is associated with a dramatic reduction in cellular proliferation and an increase in cell death. Taken together, these data suggest that the co-option of *Nkx2.5* contributes to restriction growth of the wing mesenchyme and skeletal muscle, contributing the overall reduction in wing size and function in the emu.

## Results

### Comparative expression profiling of emu and chick limb buds

The genetic alterations associated with wing reductions among the ratites are unknown. We therefore performed transcriptome analysis of chick and emu limb buds to investigate the genetic mechanisms regulating development of the vestigial emu wing. RNA sequencing (RNA-seq) analysis was performed at Hamburger and Hamilton (HH) stage 20–21^20, 21^ as a pivotal developmental time point during which the wing bud has commenced outgrowth and patterning but has not yet begun to differentiate into distinct musculoskeletal structures^22, 23^. In the absence of a reference emu genome, genes expressed in emu limb buds were assembled de novo from RNA-seq reads. To enable a cross-species comparison, gene expression profiles for emu forelimb and hindlimb were compared to identify differentially expressed genes and an analogous data set was generated for chicken. Emu and chicken fore/hindlimb differences were then analyzed and 957 transcripts were found to be significantly different between them (see Methods, Supplementary Table 1 and Supplementary Data 1). The top-ranked gene identified through this analysis (based on the fold change between the 957 genes) was *Nkx2.5*, which was strongly expressed in the emu wing bud, but not at all in emu leg bud or in either wing or leg bud in the chick. *Nkx2.5* is a homeodomain transcription factor with an essential role in development of the heart in flies, mouse, and humans^24–28^ but has no known role in limb development in any species. Expression of *Nkx2.5* in the emu wing bud is highly suggestive of an evolutionary novelty linked to developmental wing reduction.

### Analysis of *Nkx2.5* expression in diverse avian species

**Fig. 2:**
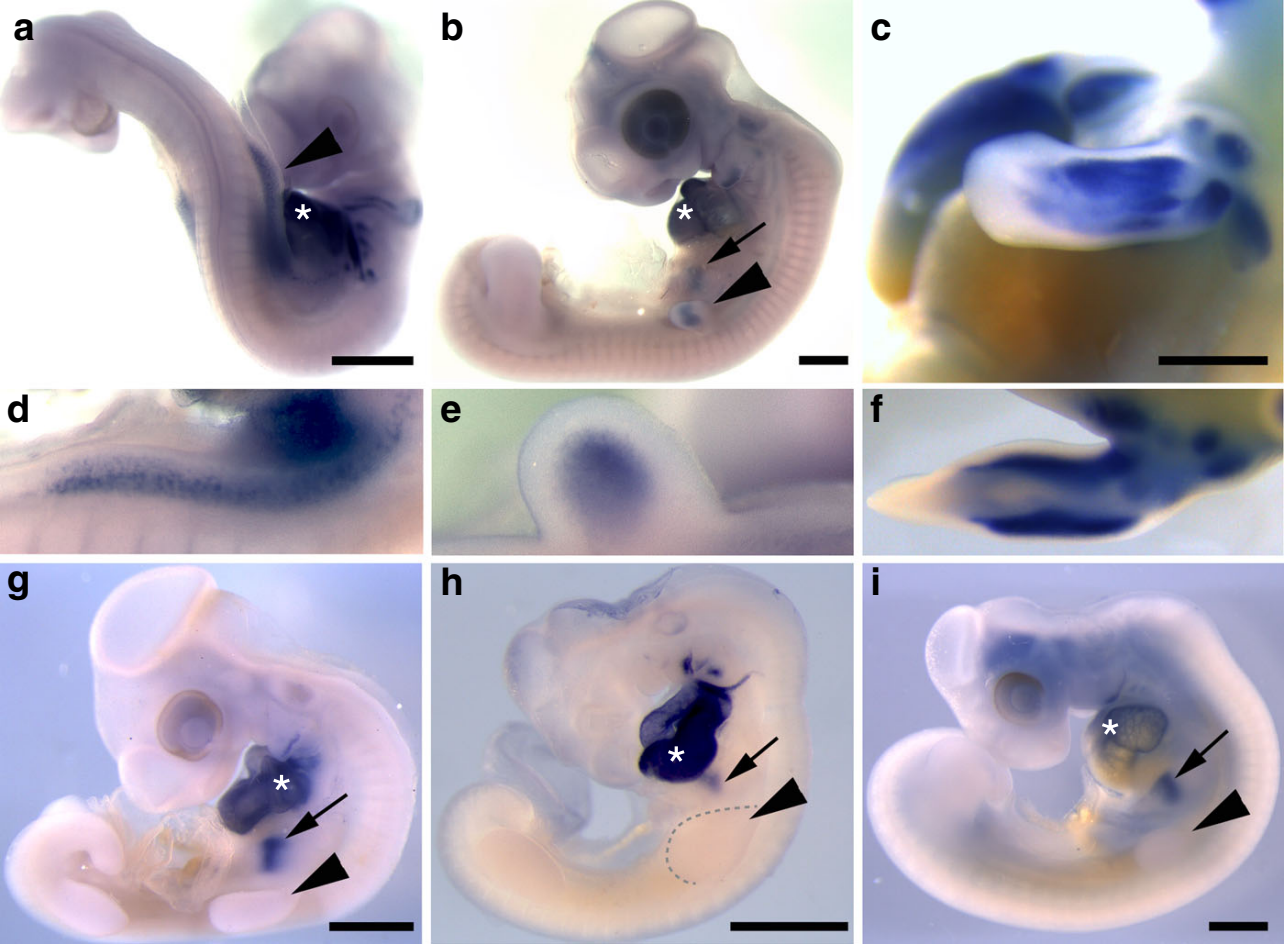
In situ hybridization analysis of *Nkx2.5* expression in avian embryos. *Nkx2.5* is expressed in the emu forelimb bud. **a**–**f** Time course of *Nkx2.5* expression in emu. **a**, **d** HH20; **b**, **e** HH25; **c**, **f** HH30; **g** chick HH22; **h** zebra finch HH22; **i** ostrich HH22. Images are representative of two or more embryos at each stage. *Arrowhead*, forelimb bud; *arrow*, stomach; *asterisk*, heart. *Scale bar* = 1 mm

**Fig. 3:**
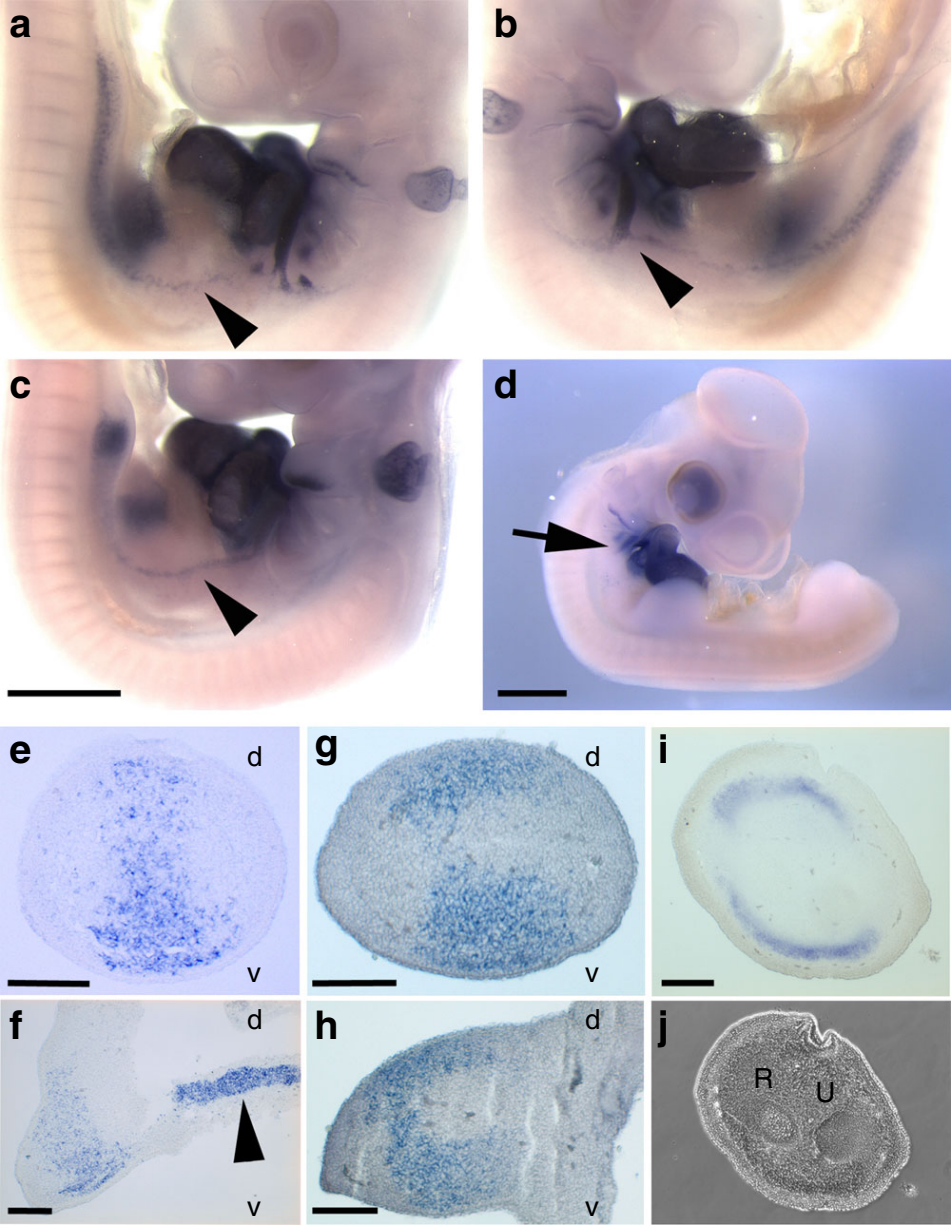
Details of *Nkx2.5* expression in the developing emu wing and surrounding tissue. **a** HH21 emu, left side, **b** HH21 emu, right side, **c** HH22 emu, left side **d** HH22 chick, right side. Note the line of *Nkx2*.5 expression extending from the 6th branchial artery and forming a continuous line of expression with the early forelimb bud (*arrowhead* in **a**–**c**). *Nkx2.5* expression is apparent in the 6th branchial artery of HH22 chick but does not extend distally to the limb bud (*arrow* in **d**). **e**–**j** Sections of emu embryos whole-mount in situ hybridization stained for *Nkx2.5*. **e** Transverse section and **f** longitudinal section of HH22 emu forelimb bud. Note the staining corresponding to the domain of expression highlighted in (**a**–**c**; *arrowhead*). **g** *Transverse section* and **h** *longitudinal section* of HH25 emu forelimb bud. **i** *Transverse section* of HH30 emu embryo and **j** corresponding phase image. *Images* are representative of two or more embryos at each stage. d, dorsal; v, ventral; R, radius; U, ulna. *Scale bar* = 1 mm (**c**, **d**), 200 μm (**e**–**i**)

To confirm the RNA-seq data and extend the analysis to additional developmental stages, we examined the distribution of *Nkx2.5* mRNA within the emu wing bud by whole-mount in situ hybridization. Expression was first observed at HH19 in the flank mesoderm preceding significant outgrowth of the wing bud, but became localized to the mesodermal core of the emerging wing primordia at the earliest stages of emu forelimb bud formation (Fig. 2a, d). As expected, *Nkx2.5* was strongly expressed in the developing emu heart and stomach, which served as positive controls. As wing bud outgrowth progressed, *Nkx2.5* expression became increasingly restricted to the superficial mesenchyme, and by HH26, when patterning of the limb has completed, became restricted to two isolated domains in the dorsal and ventral mesoderm (Figs. 2b, e and 3e–h). At HH30, *Nkx2.5* expression was strongly sustained in the dorsal and ventral limb mesenchyme in emu wing (Figs. 2c, f and 3i, j). At this stage *Nkx2.5* expression in the wing was restricted to the stylopod and zeugopod but was never observed in the autopod. In addition, a superficial line of *Nkx2.5* expression was observed extending between the developing heart and wing bud between HH 22–25 that was not present in chick (Fig. 3a–d). *Nkx2.5* was unique within the RNA-seq data set in that it was strongly and exclusively expressed within the emu wing. A similar in situ hybridization analysis of a further 15 highly ranked genes exhibiting differential expression within the RNA-seq data set excluded them from further analysis on the basis that, while there were differences in the level, distribution, or timing of expression, none showed a species-specific and wing-specific expression pattern.

We next examined expression of *Nkx2.5* in embryos of additional avian species with distinct evolutionary origins to both emu and chick. Aves consists of three major groups: (1) the Paleognathe clade harboring the ratites and tinamou, and two superorders within the Neognathe clade; (2) Galloanserae, which is made up of the gamefowl, including chicken (*Gallus gallus*), and waterfowl, and (3) Neoaves, which encompasses all other birds (Fig. 1). The ostrich (*Struthio camelus*) is broadly accepted to be basal within the Ratites^1, 4, 5, 29, 30^. Although flightless, the ostrich possesses a reduced but functional wing used in sexual display, locomotion, and fanning. The zebra finch (*Taeniopygia guttata*) is a Passeriforme representative of the Neoaves superorder with fully developed flight apparatus. In situ hybridization analysis of ostrich, zebra finch, and chick embryos indicated that *Nkx2.5* was expressed in the developing hearts, but was not expressed in the wing bud of these species (Fig. 2g–I and Supplementary Fig. 1). Thus, expression of *Nkx2.5* in the early phase of limb bud outgrowth appears to be restricted to a subset of ratites. However, in older chick and zebra finch (HH30), when individual digits have begun to form, expression of *Nkx2.5* was detected in the developing forelimb and hindlimb autopod specifically within the interphalangeal joints but was only present in the hindlimb of emu (Supplementary Fig. 2). This interphalangeal expression domain has not been reported in mice. These data suggest that *Nkx2.5* has a previously unrecognized role in development of the prospective interphalangeal joint throughout Aves.

**Fig. 4:**
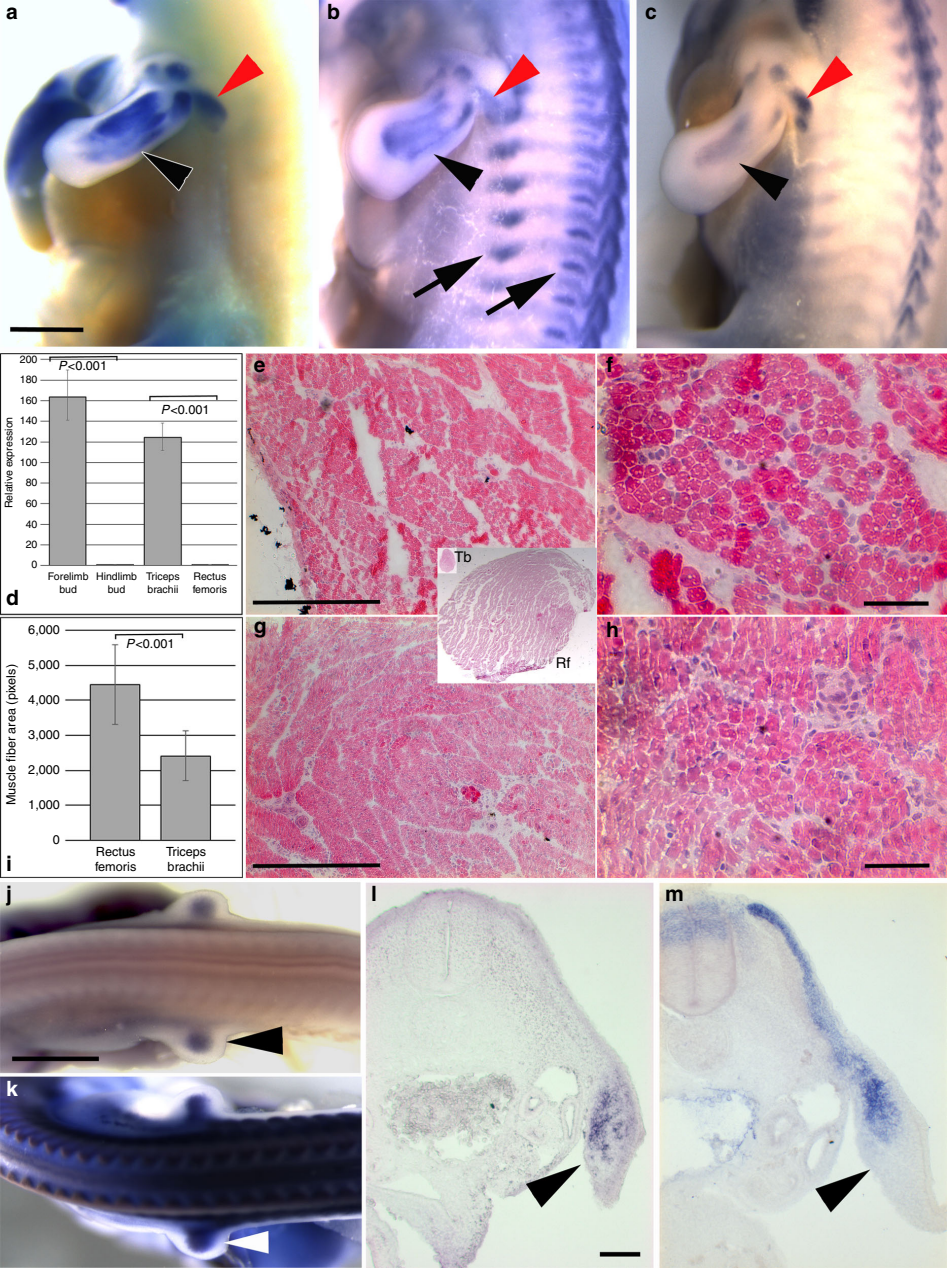
*Nkx2.5* expression in emu wing skeletal muscle. In situ hybridization analysis of muscle markers in the emu wing (**a**) *Nkx2.5*, HH30 (**b**) *Pax3*, HH30 (**c**) *Stc2*, HH30 (*n* = 2 at each stage). Note that expression of *Nkx2.5* is restricted to the wing muscle precursors (*black arrowheads* in **a**–**c**) and *latissimus dorsi* (*red arrowheads*) but is not present in axial muscle precursors (*arrows* in **b**). **d** Real-time RT-PCR analysis of *Nkx2.5* expression in embryonic and adult tissues demonstrating strong expression in the forelimb bud and Triceps brachii (mean ± SD). *Histological sections* illustrating muscle morphology in (**e**, **f**) Rectus femoris and (**g**, **h**) Triceps brachii. *Inset*, overview of the Rectus femoris (Rf) and Triceps brachii (Tb) at the same magnification, illustrating the relative sizes of the two muscles. **i** Comparison of emu muscle fiber size in leg (Rectus femoris) and wing muscle (Triceps brachii; mean area ± SD, *n* = 2). In situ hybridization analysis of markers in the wing primordia **j** *Nkx2.5*, HH23 **k** *Pax3*, HH23 **l**, *Nkx2.5* HH20, (**m**) *Pax3* HH20 (*arrowhead*, prospective wing bud). Images are representative of three embryos at each stage. Statistical significance was calculated using the Student’s *t-*test. *Scale bar* = 1 mm (**a**, **j**), 200 μm (**g**), 25 μm (**h**), 200 μm (**l**)

The expression domains of *Nkx2.5* in the emu wing bud are reminiscent of the developing muscle masses within the limb. We therefore compared the expression of *Nkx2.5* with that of the muscle markers *Pax3* and *Stc2* ^31, 32^ in emu and observed a strong overlap with the limb muscle masses and the latissimus dorsi at HH30 (Fig. 4a–c). We then examined whether the muscle-specific expression of *Nkx2.5* was maintained in mature, adult wing muscle. Comparison of *Nkx2.5* expression by qRT-PCR in HH22 wing and leg buds confirmed the specific expression within the forelimb precursor (Fig. 4d). Further, examination of adult wing muscle (Triceps brachii) compared with leg muscle (Rectus femoris) demonstrated that wing-specific expression is maintained in mature emu muscle (Fig. 4d). Ectopic expression of *NKX2.5* in skeletal muscle is associated with myotonic dystrophy type 1 in humans and results in a distinctive histological presentation of muscle, including central nuclei indicative of active muscle repair^33, 34^. Histological examination of late fetal muscle in the emu did not reveal any major difference in the overall appearance of wing and leg muscle such as central nuclei, but examination of individual muscle fibers revealed a marked reduction in fiber diameter in wing muscle relative to leg muscle (Fig. 4e–h).

### Muscle-specific mis-expression of *Nkx2.5*

In vertebrate embryos, limb skeletal muscle cell progenitors derive from the somites, with precursor cells undergoing an epithelial-to-mesenchyme transition and migrating from the ventrolateral lip of the dermomyotome into the limb buds as *Pax3* and *Pax7*-positive mesenchyme^35^. The early expression of *Nkx2.5* in the emu forelimb bud may therefore be due to immigrating myogenic precursors from the somites, or it may be derived from resident lateral plate-derived mesenchyme (or both). To address this point, we examined the relative timing of *Nkx2.5* expression and immigration of myogenic progenitors, marked by *Pax3*. *Nkx2.5* mRNA expression strongly overlaps with *Pax3* in the limb bud (Fig. 4j, k) but is expressed in a domain partially overlapping and adjacent to *Pax3* + immigrating myogenic progenitors at earlier stages (Fig. 4l, m), indicating that *Nkx2.5* is already expressed in resident (lateral-plate-derived) mesenchymal cells. In addition, we have recently demonstrated reduced cell proliferation associated with retarded emu forelimb bud development associated with resident mesenchyme but not *Pax7* + immigrating cells^19^, implicating resident mesenchymal cells as the cell type likely involved in development of the reduced emu wing bud.

**Fig. 5:**
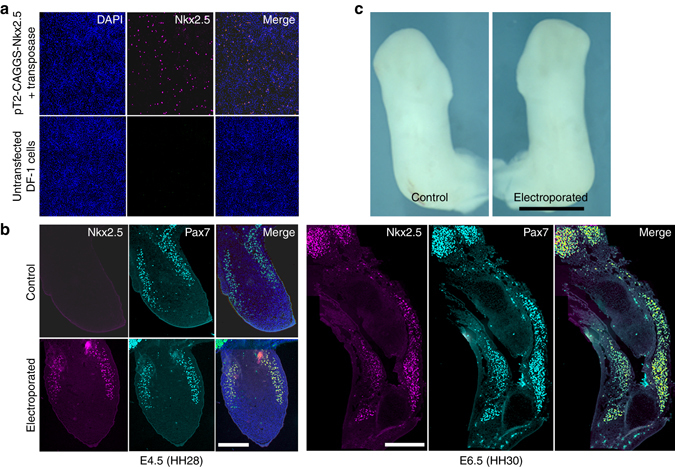
Mis-expression of Nkx2.5 in somites and developing muscle. **a** Demonstration of Nkx2.5 expression in chicken DF1 fibroblasts using pT2-CAGGS-Nkx2.5 detected by anti-Nkx2.5 immunostaining. **b** Co-expression of Nkx2.5 and Pax7 in somite-derived muscle precursors in chick limb buds and limbs. **c** Electroporated limb demonstrating normal phenotype (*n* = 4). *Scale bar* = 250 μm (**b**), 1 mm (**c**)

However, to assess its potential effect in somite-derived muscle precursors, we overexpressed *Nkx 2.5* specifically in somites of chicken embryos at E2.5 (HH stage 16–17), by electroporating TOL2 expression plasmids into the ventrolateral lip of somites 16–20 (which generate the forelimb musculature)^36^. In the presence of co-electroporated transposase, TOL2 integrates into the genome, and is hence expressed in all daughter cells^37^. TOL2.GFP electroporations or unelectroporated contralateral limbs served as controls. Overexpression of Nkx 2.5 in somite progenitors resulted in co-expression of Nkx2.5 and the myogenic marker Pax7 but no overt effect upon limb development or morphology (Fig. 5). Taken together, these data indicate that the effects of *Nkx2.5* in the retarded emu wing bud are unlikely to derive from direct cell autonomous effects in somite-derived limb muscle progenitors.

### Mis-expression of Nkx2.5 throughout the limb bud

**Fig. 6:**
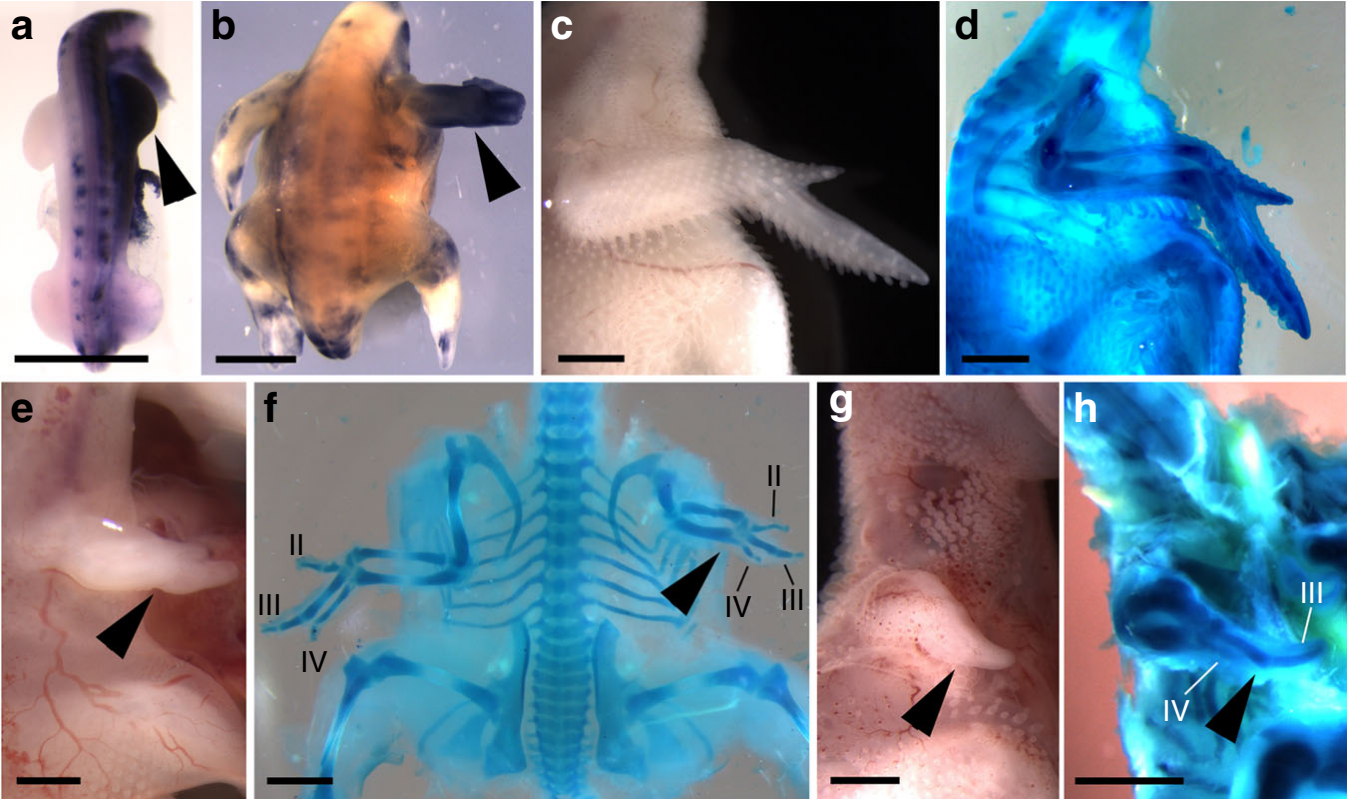
Impact of *Nkx2.5* mis-expression throughout chick wing. **a**, **b** In situ hybridization for *Nkx2.5* demonstrating viral mis-expression (*arrowhead*) in **a** the right wing bud 2 days post infection and **b** 4 days post infection. **c**, **d** control virus infection demonstrating unaltered wing morphology in (**c**) unfixed and **d** alcian blue-stained preparation of an embryo harvested 10 days after infection (*n* = 6). **e**–**h** *Nkx2.5*-expressing virus infection, **e** unfixed, and **f** alcian blue-stained preparation (*n* = 19) of an embryo harvested 6 days after infection demonstrating reduced wing growth along the entire proximodistal axis of the right wing (*arrowhead*) in comparison to uninfected left hand side, **g** unfixed, and **h** alcian blue-stained preparation of an embryo harvested 10 days after infection demonstrating extreme reduction of wing growth and digit loss (*arrowhead*). *Scale bar* = 2 mm

We next investigated the potential of *Nkx2.5* to have an impact on wing morphology following mis-expression throughout the entire developing chick wing using the RCASBP avian retrovirus^38, 39^. Unlike the TOL2 vector, the RCASBP viral vector can spread to all cells following initial infection. Infection of right side lateral plate mesoderm at HH10–12 resulted in widespread unilateral *Nkx2*.5 mis-expression throughout the limb by HH21, 2 days after infection, and expression was maintained throughout development (Fig. 6a, b). Limb buds subjected to *Nkx2.5* mis-expression at this stage exhibited reduced outgrowth that distinguished them from contralateral uninfected left limb buds (Fig. 6a, b and Supplementary Fig. 5). Embryos infected with a control virus not harboring any exogenous gene exhibited comparable morphology between infected and contralateral wings (Fig. 6c, d). At the digit condensation stage, *Nkx2.5* expression resulted in a reduced autopod and a reduction in the size of the digit II and IV condensations with a relative sparing of the digit III condensation, similar to the configuration observed in the emu autopod (Supplementary Fig. 4). As development proceeded, *Nkx2.5* mis-expressing chicken wings became progressively more growth-retarded relative to controls and exhibited a variety of abnormal phenotypes ranging from a reduction of the autopod in the anterioposterior axis, a marked reduction in growth along the entire proximodistal axis, to production of a dramatically reduced and vestigial wing-like structure (Fig. 6e, f). Analysis of older specimens revealed reduced growth of all skeletal elements and, in extreme cases, a reduction of the autopod skeleton to a single medial digit in wings mis-expressing *Nkx2.5*, analogous to that in emu embryos (Fig. 6g, h). Even though the timing and extent of infection using this retroviral system are inherently variable, analysis of *Nkx2.5*-mis-expressing embryos over a range of developmental stages resulted in visibly reduced wing buds or wings in 77.2% (*n* = 217/281) of cases (Supplementary Fig. 5).

### Expression profiling of Nkx2.5 mis-expressing limb buds

**Fig. 7:**
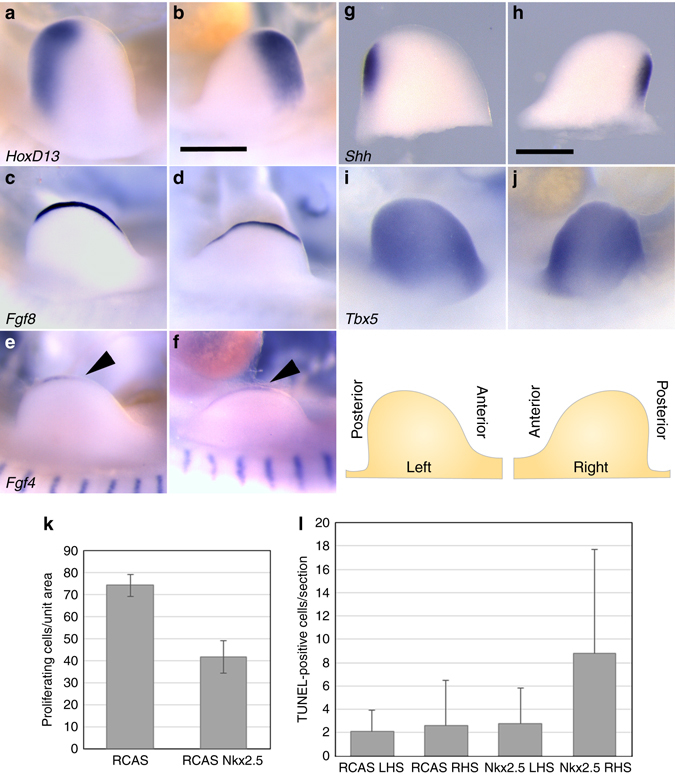
Impact of *Nkx2.5* mis-expression on expression of essential limb-patterning genes. In situ hybridization of forelimb buds 2 days after infecting the right wing bud with *Nkx2.5*-expressing virus. **a**, **c**, **e**, **g**, **i** left (uninfected) and **b**, **d**, **f**, **h**, **j** right (infected) forelimb buds of the same embryo. **a**, **b** *HoxD13*, **c**, **d** *Fgf8*, **e**, **f** *Fgf4*, **g**, **h** *Shh*, **i**, **j** *Tbx5* (*n* = 3 for HoxD13, Fgf4 and 8, *n* = 9 for Shh). Orientation of limb buds is as illustrated *bottom right*. **k** *Nkx2.5* expression reduces proliferation in limb bud mesenchyme. Control limb bud mesenchyme possesses 74.1 ± 4.8 (mean ± SD, *n* = 3) proliferating cells per unit area, while *Nkx2.5*-expressing limb buds have 41.7 ± 7.3 proliferating cells per unit area (*n* = 5). **l** Cell death in response to Nkx2.5 mis-expression in chick limb buds (mean ± SD, *n* = 3 for control and Nkx2.5-expressing embryos). Statistical significance calculated using Student’s *t*-test. *Scale bars* = 0.5 mm

The molecular consequences of *Nkx2.5* co-option to the limb bud are unclear since the known roles of *Nkx2.5* entail regulation of cardiac, thyroid, and stomach development^28, 40, 41^. We investigated the impact of *Nkx2.5* expression within the wing primordia on gene expression profile using RNA-seq analysis of individual chick wing buds mis-expressing *Nkx2.5* at HH21, 2 days after infection. A total of 26 genes including *Nkx2.5* were observed to exhibit statistically significant differential expression between control and *Nkx2.5*-mis-expressing wing buds (Supplementary Data 2 and Supplementary Table 2). None of these genes have known roles in regulating limb morphogenesis or growth or are known targets of Nkx2.5, suggesting that *Nkx2.5* alters wing growth using novel regulatory strategies. An analysis of key genes with established roles in growth and patterning of the limb buds in this data set revealed no significant differential expression between controls and *Nkx2.5* mis-expressing limb buds (Fig. 7, Supplementary Data 2 and Supplementary Table 3). Consistent with the dramatically reduced wing size, *Nkx2.5* mis-expression in the chick wing bud resulted in a 44% reduction (*p* ⩽ 0.001) in cellular proliferation relative to control embryos with a substantial but highly variable increase in cell death throughout the limb bud mesenchyme (Fig. 7).

## Discussion

The loss of flight in the ratites is accompanied by numerous anatomical changes including increased body size, loss of the sternal keel,and a reduction in wing size. The embryological origins of these changes are poorly characterized, and few genetic clues to explain these phenomena have been revealed. We compared gene expression profiles in the early limb bud of chick and emu embryos to identify genes that were differentially expressed between the two species that would provide insight into the unique developmental program resulting in the reduced emu wing. The cardiac transcription factor *Nkx2.5* was strongly expressed in the emu wing bud but was absent from the chick wing bud. Examination of Nkx2.5 expression in the flighted zebra finch and ostrich, another ratite, during early limb bud stages did not reveal any *Nkx2.5* expression. Zebra finch, chicken, ostrich, and emu are collectively representative of the three key nodes across the avian family tree (Fig. 1), suggesting that *Nkx2.5* expression in the developing wing bud is a rare phenomenon and may be unique to the emu and its closest relatives. Co-option of existing genes to novel expression domains during development has been previously associated with the acquisition of new anatomical traits^42^. Wing reduction and the loss of flight may appear at odds with the concept of acquisition of new traits. However, maintenance of anatomical structures required for flight is interpreted as being evolutionarily costly^43^ and, when viewed in the context of acquisition of gigantism, wing reduction becomes a feature of a new trait in the broader sense.

The extant ratites comprise the ostrich (the basal member of the ratites), emu, kiwi, cassowary, and rhea, and, together with the tinamou, are grouped into the Paleognathe clade. Rhea, like ostrich, has a reduced but functional wing harboring a tridactylous autopod^12^, while tinamou possess a wing morphology comparable to other flighted birds. It is therefore unlikely that *Nkx2.5* is expressed in the wing bud of either Rhea or Tinamou. The emu, kiwi, and cassowary are currently considered to form a monophyletic clade^1^, and cassowary and kiwi harbor vestigial wings, broadly similar in morphology to emu, suggesting that both kiwi and cassowary may also express *Nkx2.5* in the wing bud. However, cassowary and kiwi are both endangered species, and it has not been possible to obtain embryos to test this hypothesis. Thus, *Nkx2.5* expression in the wing bud may have arisen in the most recent common ancestor of the kiwi–emu–cassowary clade around 78 Ma, or as recently as 27 Ma when the *Dromaiidae* and *Cassuariidae* diverged. Identification of *Nkx2.5* expression in the embryonic wing of emu but not ostrich demonstrates that there are at least two distinct developmental trajectories associated with wing reduction, consistent with a polyphyletic origin of ratites as previously suggested^4, 5, 44^.

In addition to expression within the limb bud mesenchyme, examination of older emu embryos revealed *Nkx2.5* expression in skeletal muscle precursors, maturing muscle, and in the interphalangeal joints. The emu wing muscles are diminutive, and histological examination of wing muscle (Triceps brachii) revealed reduced muscle fiber diameter in comparison with hindlimb muscle (Rectus femoris). The presence of novel, tightly regulated domains of *Nkx2.5* expression in the emu wing bud and muscle prompted an examination of the developmental role for *Nkx2.5* on wing morphology. Induced expression of *Nkx2.5* has been shown to reduce muscle cell differentiation and myotube formation in vitro^34^, suggesting the potential for a direct role for *Nkx2.5* in inhibiting the growth of wing muscle in the emu. However, we observed no reduction in muscle development or limb outgrowth following a restricted mis-expression of *Nkx2.5* in somitic myogenic precursors in chick. The overall morphology of the limbs is regulated by the collective influence of patterning factors distributed in all three axes. While the skeletal and muscle precursors migrate into the developing limb bud in parallel and their distribution is influenced by a number of the same factors^45, 46^, the ability of developing muscle to influence the final form of skeletal elements appears restricted. This is in keeping with previous studies in which experimental ablation of skeletal muscle did not result in altered limb morphology^47, 48^ and the association of *Nkx2.5* expression with emu wing muscle likely does not strongly influence overall wing morphology. In contrast, a broader, viral mis-expression in chick did result in a significant reduction in limb bud and limb outgrowth producing reduced skeletal elements and, in extreme case, digit loss, consistent with previous data demonstrating the essential role of early patterning influences on later limb morphology^10^.

While neither of the functional studies reported here precisely recapitulates the endogenous emu wing *Nkx2.5* expression pattern, these data show that mis-expression of *Nkx 2.5* within the chicken forelimb buds results in a strong growth reduction that can produce small emu-like wings. As we did not observe the same effect when *Nkx 2.5* overexpression was restricted to the somite-derived muscle cell progenitors, we conclude that the effects of *Nkx 2.5* are mediated via lateral-plate-derived mesodermal cells. Although we exclude a direct effect of *Nkx 2.5* in immigrating muscle cell precursors, *Nkx 2.5* nevertheless shows an intriguing muscle-type expression pattern at later developmental stages (Fig. 4). It is possible that expression of the gene shifts from lateral plate-derived mesoderm into muscle cell progenitors once they have colonized the limb bud. An alternative is that *Nkx 2.5* is expressed in lateral plate mesenchymal cells but becomes progressively restricted to a subpopulation of cells that show a similar expression domain to that of muscle cells. Consistent with this hypothesis, the limb buds of RCAS *Nkx2.5*-infected embryos are reduced in size from the time they begin to grow out from the body wall (Fig. 6 and Supplementary Fig. 3). One such group of cells could be connective tissue cell progenitors, which derive from the lateral plate mesoderm but have a striking muscle-type arrangement. Indeed, these *Tcf4*-positive connective tissue precursors have been found to influence patterning of limb musculature, at least in the chicken limb bud^49^. However, we found that *Nkx 2.5* and *Tcf4* are expressed in different domains in the emu forelimb bud (Supplementary Fig. 5). The expression pattern of *Tcf4* in the emu wing appears to be restricted to a superficial location in the early limb bud in contrast to the more central location reported in the chick^49^. The significance of this observation is uncertain and will require further analysis to clarify, but raises the possibility that the interaction between the connective tissue and myogenic precursors varies in the chick and emu wings. It is therefore possible that the effects of *Nkx 2.5* on retarded growth of the wing in emu may be mediated via a novel subpopulation of mesenchymal cells or that differences in the distribution of known population are so sufficiently altered in emu that they cannot be identified using established markers. This functional assessment of the potential for *Nkx2.5* expression to reduce growth in the developing wing demonstrates that co-option of *Nkx2.5* to the emu wing primordia is likely an important factor contributing to the altered morphology in the adult emu wing, although further work will be required to demonstrate this definitively.

Our RNA-seq analysis revealed over 900 expression differences between chick and emu limb buds. It is therefore highly unlikely that alteration in any single gene accounts for all the morphological variation between the wings of emu and other avian species. The previously identified delay in initiation of *Tbx5* expression^18^ may contribute to the late onset of wing bud outgrowth in the emu. However, we have observed robust *Tbx5* expression in emu embryos from the onset of wing bud development (Supplementary Fig. 6), and it is not clear whether delayed expression of *Tbx5* in the lateral plate mesoderm contributes to the diminutive size of the wing or is a consequence of a reduced wing bud field. Similarly, the reduced wing bud relative to the leg bud expression of *Shh* we have observed in emu^19^ may contribute to reduction in the anterioposterior growth of the forelimb autopod or may reflect a reduced requirement for *Shh* within the smaller wing bud field. In addition, our expression profiling was performed at a single time point, soon after initiation of wing bud outgrowth. It is possible that there are additional significant differences in timing or level of other key factors prior to limb bud initiation that were not apparent from our study. The relative contributions to the morphology of the mature wing from the complex array of genes exhibiting expression differences in the emu relative to chick await further investigation.

The ability of virally mis-expressed *Nkx2.5* to alter limb growth and patterning in chick prompted an examination of gene expression consequences. Despite the dramatic effect of *Nkx2.5* mis-expression on limb morphology, we did not detect any disruption to the known limb-patterning network. However, there was a strong reduction in cellular proliferation within *Nkx2.5*-expressing limb buds, suggesting that the major influence on limb morphology may be through growth restriction. This is consistent with the reduced cell proliferation we recently reported in the developing emu wing^19^. Furthermore, in mouse embryos, ectopic expression of *Nkx2.5* in the prospective sinoatrial node within the developing heart, where it is normally repressed, results in a marked reduction in cellular proliferation^50, 51^, supporting the ability of Nkx2.5 to repress tissue growth. We also detected a highly variable increase in cell death in *Nkx2.5*-mis-expressing limb buds. Given the consistent phenotype resulting from *Nkx2.5* mis-expression, it is difficult to determine whether loss of cells through apoptosis is a driver or consequence of the altered phenotype. However, in combination with reduced proliferation, even a variable level of cell death is likely to result in reduced limb bud size and compromise the precursor pool available for limb outgrowth. The emu wing is dramatically reduced relative to overall body size at all stages, and the restricted availability of limb progenitors may be a major driver of reduced tissue expansion in both the proximodistal and anterioposterior axes resulting in the observed morphological changes. Thus, *Nkx2.5* appears to have been superimposed on the known limb regulatory network and has the ability to override or modulate the normal patterning mechanisms, producing emu-like growth restriction in the chick wing in the presence of a fully functional limb-patterning apparatus.

Taken together, these data support the hypothesis that the novel co-option of the transcription factor *Nkx2.5* into the limb developmental program was a significant event in the wing reduction specifically observed in emu. This study further supports a polyphyletic origin for the flightless ratites, as *Nkx2.5* is not expressed in ostrich forelimb buds. Experimental mis-expression within the developing chick wing demonstrates the ability of *Nkx2.5* to inhibit growth within the wing field, while the observation of sustained expression within the developing and mature emu wing muscle suggests that this growth restriction in emu embryos is also directed toward the myogenic lineage. We hypothesize a dual role for *Nkx2.5* in the emu forelimb bud: restriction of growth in the early limb bud and later restriction of muscle differentiation, contributing to the vestigial wing phenotype seen in adult birds.

Future analysis should now focus on the regulation of emu *Nkx2.5* gene. The novel domain of *Nkx2.5* expression in the emu wing bud suggests an alteration to non-coding regulatory elements, and, while the timing of this genetic novelty within the ratite lineage remains to be defined, phylogenetic data suggest that co-option of *Nkx2.5* may correspond to the most recent common ancestor of the kiwi–emu–cassowary clade. However, developmental studies are restricted to extant species from which viable embryos are accessible. Identification of the genomic alterations resulting in the novel limb domain of *Nkx2.5* expression in emu would allow examination of related regulatory sequences in additional species for which embryonic material is unavailable, including extinct species. While there are no direct data available on the nature of these alterations, there are a number of points to consider. *Tbx5* is essential for forelimb development and is expressed in a forelimb-restricted domain in all species examined, including emu^15, 18, 52, 53^. This makes *Tbx5* (or factors controlling forelimb-specific expression of *Tbx5*) a compelling candidate as a regulator of *Nkx2.5* expression in the emu wing domain. Similarly, *Pax3* is essential for development of the hypaxial limb and tongue musculature, and a hypaxial *Pax3* enhancer has been identified^54^, demonstrating a distinct regulatory mechanism controlling epaxial and hypaxial muscle development. Interestingly, the lingual myoblasts transiently express *Nkx2.5* ^55, 56^, raising the possibility that the co-option of *Nkx2.5* to the developing wing may involve a forelimb-specific modification to a hypaxial musculature regulatory module in emu. In addition, we have identified a novel pan-avian domain of *Nkx2.5* within the developing interphalangeal joints (Supplementary Fig. 2), suggesting the existence of a limb-oriented regulatory module in birds which may also have contributed to expanded *Nkx2.5* expression in the emu wing. The relative contribution from regulatory elements for any of these sites to co-option of *Nkx2.5* in the emu wing from the earliest stages of development is yet to be determined. However, identification of the genomic alterations associated with wing-specific expression of *Nkx2.5* in the emu will be useful in clarifying a number of remaining contentious issues including the unresolved question of vicariant or flighted dispersal of the kiwi ancestor to New Zealand. In addition, refining the timing of *Nkx2.5* co-option will also address the question of whether *Nkx2.5* is a driver or a downstream consequence of the evolutionary cascade that has led to flightlessness in this ratite.

## Methods

### RNA extraction and sequencing

Duplicate pools of HH stage 20–21 (day 8) emu embryo forelimb and hindlimb buds were harvested and stored at –80 °C prior to processing. Each of the four pools comprised seven pairs of fore or hindlimb buds. Total RNA was extracted using the RNeasy micro kit (QIAGEN), with on-column DNAsing to remove contaminating genomic DNA. Integrity of the total RNA (3 μg) was first confirmed on a bioanalzyer. The four RNA replicates were then poly A-selected, reverse-transcribed, fragmented, bar-coded, and sequenced using the Illumina HiSeq2000 at Australian Genome Research Facility (AGRF) in Melbourne, to a depth of approximately forty million 100 bp paired-end reads. Samples in the *Nkx2.5* mis-expression experiment were sequenced to a depth of ~120 million 76 bp paired-end reads on a Next-Seq 500 at the Murdoch Childrens Research Institute translational genomics unit.

RNA-Seq reads were trimmed to remove low confidence base calls at the beginning and end of reads using trimmomatic^57^ (with parameters “LEADING:20 TRAILING:20 MINLEN:50”). Trimmed reads were used for all subsequent data analysis steps.

### Analysis of RNA-seq from emu and chicken embryonic limbs

In order to determine which genes had different levels of forelimb to hind limb expression between species, we first quantitated expression level counts from chick and emu separately and then matched the annotation between species before statistical tests for differential expression were performed. Specifically, chick reads were mapped to the chicken reference genome, galGal4, using tophat version v2.0.6^58^. The chicken Ensembl^59^ annotation was downloaded from UCSC table browser^60^ (version available on 8th November 2013) and passed to tophat using the option, “-G”. The number of read pairs–*counts*–aligning to each Ensembl gene was calculated using featureCounts^61^.

Because there is no public reference genome for emu, we de novo-assembled pooled emu reads using Trinity version r2013_08_14^62^. The assembly produced 424,913 contiguous sequences (contigs), representing complete or partially expressed emu transcripts. To determine gene expression levels, reads were mapped back to contigs using bowtie v0.12.7 (with parameters “--all -X 1000”). Mapped reads were processed with Corset version 0.94^63^, which groups assembled contigs into gene-level loci and counts the number of reads aligning to each. Emu forelimb or hindlimb sample status was passed to Corset with the parameter, “–g”. Corset reported 89,174 gene-level loci.

Next, emu loci were annotated with chicken Ensembl gene IDs. This was achieved by first aligning emu contigs against chicken Ensembl transcripts using BLASTX 2.2.25^64^. Alignments with an *e*-value of 10^−5^ or more were discarded. Contigs were annotated with the Ensembl ID of the best match based on *e*-value. Emu loci were annotated with the chick Ensembl IDs of their constituent contigs. In all, 17,768 Emu loci could be annotated. For 699 of these, multiple Ensembl genes matched. These loci were discarded from further analysis because assigning counts to genes was ambiguous. We aggregated the counts for emu loci annotated to the same Ensembl ID. As a result, 11,372 genes had both emu and chicken count data and were subsequently tested for differential expression.

Emu and chicken counts were analyzed together using voom^65^, with a model containing factors for each of emu forelimb, emu hindlimb, chicken forelimb, and chicken hindlimb (denoted EF, EH, CF, and CH, respectively). We then tested for a difference in the ratio of the expression of emu forelimb/emu hindlimb compared to chicken forelimb/chicken hindlimb, using the contrast, (EF-EH) - (CF-CH). Note that chicken and emu counts cannot be directly compared (e.g., with the contrast EF–CF), due to species-specific biases such as quality of the assembled emu transcriptome. In all, 957 genes were significantly differentially expressed (false discovery rate (FDR)-adjusted *P*-value < 0.05^66^). Next, to identify genes dominated by differential expression in the emus, we ranked the significantly differentially expressed genes in descending order of |log2(emu fore/hind fold change)| − |log2(chicken fore-/hind limb fold change)| i.e., the change in emu is larger than the change in chicken. *Nkx2.5* was the top-ranked gene according to this scheme.

### Analysis of RNA-Seq from ***NKX2.5*** mis-expression in chicken wing

RNA was extracted from individual control RCAS or RCAS *Nkx2.5* infected wing buds 2 days after infection (HH22). Samples were screened for *Nkx2.5* expression by qPCR (*Nkx2.5* group only) and RNA-seq performed on RNA from three individual embryos per group. RNA-Seq reads were mapped and counted using the same method as for the chicken reads in the emu-chicken analysis. Only genes with ten or more counts in two or more samples were analyzed in voom (13,684 genes). We modeled the data with a coefficient for *Nkx2.5* mis-expression and for sex, where sex was determined using counts from the female specific gene *HINTW*. 10 genes were significantly differentially expressed between the *Nkx2.5* mis-expression and control groups (FDR adjusted *P*-value < 0.05). *Nkx2.5* was the top ranked gene, confirming its mis-expression compared to controls.

### In situ hybridization

In situ hybridization was performed using a standard protocol available from the GEISHA website (http://geisha.arizona.edu/geisha/). An in situ probe to emu *Nkx2.5* was constructed (Genscript, USA) against the following sequence assembled from RNA-seq data and was used in all avian species in this study.

GAGAGACCATCTAGCAAACGTCCTAAAGCTCACCTCTACCCAGGTTAAAATTTGGTTCCAGAATAGAAGGTATAAATGCAAAAGGCAGAGACAGGATCAGACCCTGGAAATGGTGGGGATCCCGCCGCCGCGGCGGATAGCGGTGCCCGTGCTGGTGCGCGATGGGAAGCCCTGCTTGGGGGAGTCTTCTCCCTACAGTTCGCCCTACAATGTCAGCATCAACCCCTACAGCTACAACGCCTACCCCGCGTACACCAACTACAACAGCCCCGCCTGCAACGCCAACTACAACTGCAACTACCCCTC CATGCAGACCATGCAGCCCTCCGCGGCCGGCAACAACTTC ATGAACTTCGGCGTGGGGGACTT GAACGCGGTGCAGAC GCCCATCC

In situ hybridization probes were constructed (Genscript, USA) for chick Fgf4 (nucleotide 186–546 of Genbank sequence U14654.1) and HoxD13 (nucleotide 1505–1908 of NCBI refseq NM_205434.1). The Tbx5 probe was cloned by PCR (NCBI AF069396 nucleotide 1825–2144), and the probes for Fgf8 and Shh were gifts from Sangwei Lu and Philippa Frances-West, respectively. Images were captured on a Leica M60 dissecting microscope using Leica LAS v4 software.

### RCAS ***Nkx2.5*** construction and chick infection

The coding sequence of chick *Nkx2.5* (nucleotide 211–1167 NCBI Refseq NM_008700.2) was synthesized and cloned into the ClaI site of RCASBP(A)^39^ (Genscript, USA). Virus stocks were prepared and injected into the right hand side lateral plate mesoderm of HH10-12 chick embryos as previously described^38^. Fertilized specified pathogen-free eggs were obtained from SPAFAS Australia (Woodend, Victoria).

### Muscle RNA extraction, qRT-PCR and histology

RNA was extracted from frozen adult muscle using Trizol (Life Technologies, USA) and 1 μg of total RNA was reverse-transcribed using random priming. Quantitative PCR was performed using Lightcycler SYBR green master mix (Roche Diagnostics, GmbH) using Nkx2.5 primers F:CAAGGCGGACAAGAAAGAAC R: GACTTGGGCTTGAGAAAAGAG and normalized using the geometric mean of Ct values for the U6 and Map3k genes. Late fetal emu muscle (~E45) was frozen in liquid nitrogen, sectioned at 10 μm thickness, and processed for standard hematoxylin and eosin staining. Muscles from two emu specimens were examined. Fiber areas were determined using the pixel counting tool in Photoshop on five non-consecutive sections for each muscle and are expressed as the mean ± SD with statistical significance calculated using a two-tailed Student’s *t*-test.

### Proliferation and cell death assays

The impact of *Nkx2.5* expression on limb bud cell proliferation was examined using EdU metabolic labeling and manual counting of labeled nuclei. Chick embryos were EdU-labeled by pipetting 400 μl of 500 μM EdU directly onto embryos and incubation for a further 4 h^67^ 2 days after infection with control RCAS virus or RCAS *Nkx2.5* virus. Frozen sections (10 μm) were processed to reveal EdU incorporation as per the manufacturer’s protocol (Click-iT EdU Alexa 488 labeling protocol, ThermoFisher, USA). Cell death was examined using TUNEL staining as per the manufacturer’s protocol (Roche Diagnostics, GmbH). Sections were viewed on an Olympus IX-70 microscope at ×20 magnification and images captured on a Media Cybernetics Evolution VF monochrome camera using QCapture Pro software resulting in a standard 117.8 × 88.1 mm image. A 20 × 20 mm square was calibrated to enclose 50–100 nuclei in control limb bud images and this unit square was copied onto all images. Nuclei were counted manually in sections from three control and five *Nkx2.5*-expressing embryos. Data reported are pooled from all embryos examined. Cell counts are expressed as mean ± SD. Statistical significance was calculated using a two-tailed Student’s *t*-test.

All experiments were performed in accordance with the guidelines of the Animal Ethics Committee of the Murdoch Children’s Research Institute. Fertilized emu eggs were purchased from a commercial production facility (Emu Logic, Toorahweenah, NSW) and no eggs from wild emu were used in this study.

### Code availability

The code used to analyze the RNA-seq data generated as part of this study is available at https://github.com/Oshlack/Farlie_emu_wing_RNASeq_analysis.

RNA-seq data sets that support the findings of this study are deposited at SRA under accession code SRP106587.

### Data availability

The authors declare that all data supporting the findings of this study are available within the article and its Supplementary Information files or from the corresponding author upon reasonable request.

## Electronic supplementary material

Supplementary Information

Supplementary Data 1

Supplementary Data 2
