# Supplementary material for: Co-option of the cardiac transcription factor Nkx2.5 during development of the emu wing
Source: Nat Commun. 2017 Jul 25;8:132. doi: 10.1038/s41467-017-00112-7 (PMC5526984; doi:10.1038/s41467-017-00112-7)
Supplement: Supplementary file 1 — Supplementary Information [file 41467_2017_112_MOESM1_ESM.pdf]

Title of file for HTML: Supplementary Information
Description: Supplementary Figures, Supplementary Tables.

Title of file for HTML: Supplementary Data 1
Description: Emu and chicken fore/hindlimb expression differences.

Title of file for HTML: Supplementary Data 2
Description: RNA-seq analysis of control vs Nkx2.5 expressing chick limb buds.

Supplementary Information

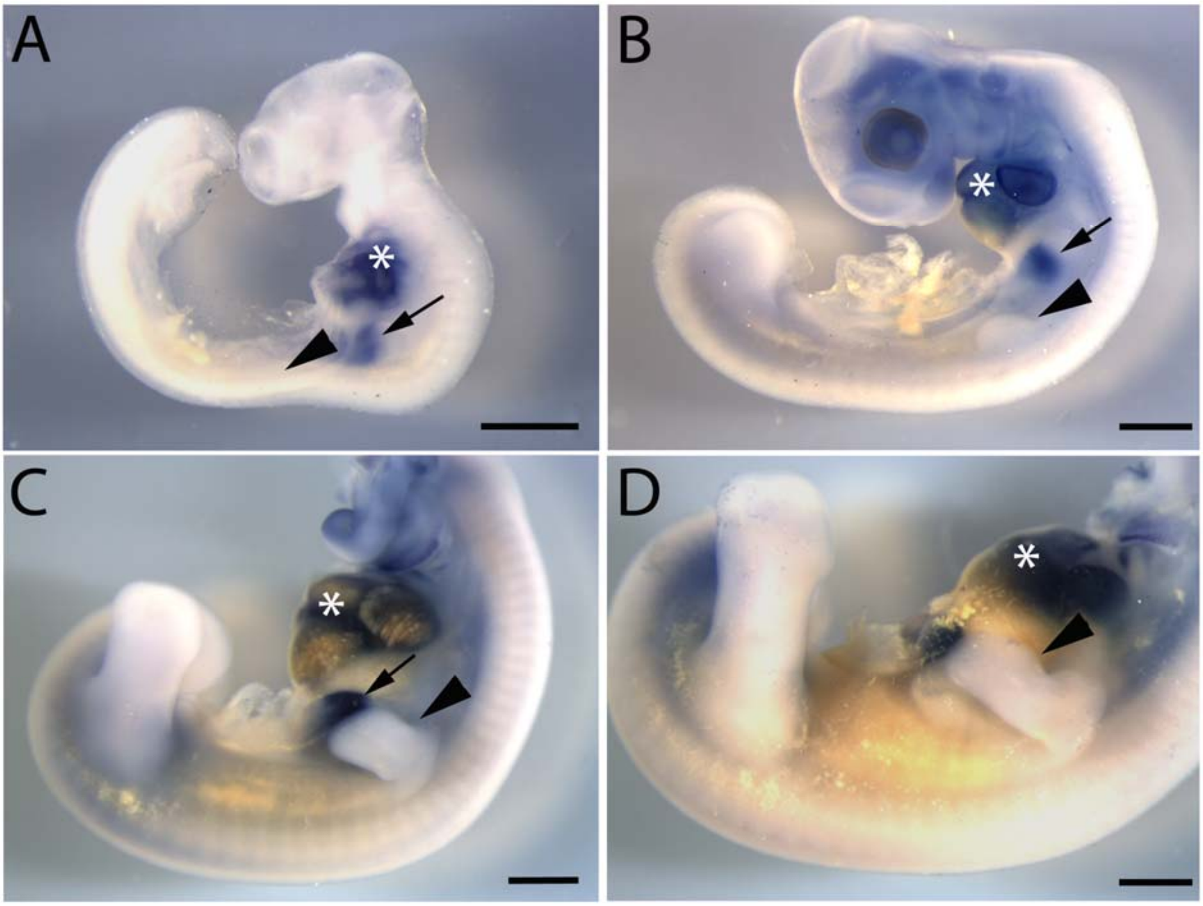

Supplementary Figure 1: Extended data illustrating expression of *Nkx2.5* within a developmental series of ostrich embryos. (A) HH21, (B) HH24, (C) HH28, (D) HH30. Images are representative of at least 2 embryos at each stage. Asterisk, heart; arrow, stomach; arrowhead, forelimb. Scale bar = 1 mm.

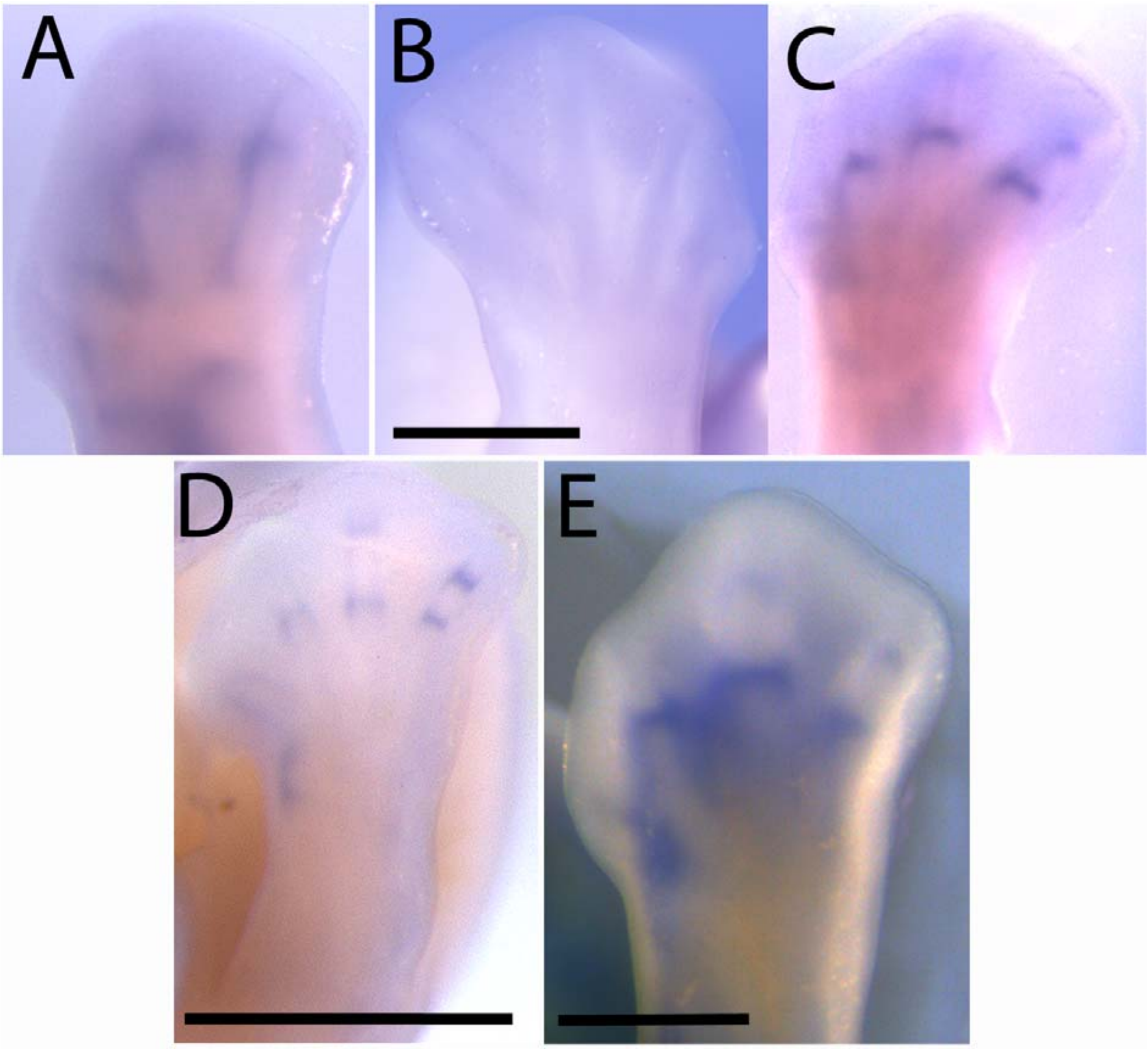

Supplementary Figure 2: *Nkx2.5* expression associated with phalangeal joints. (A) chick HH30 forelimb autopod, (B) chick HH30 sense control (C) chick HH30 hindlimb autopod, (D) zebra finch HH30 hindlimb autopod, (E) emu HH30 hindlimb autopod. (n = 3 for chick and emu, n = 2 for zebra finch) Scale bar = 1 mm.

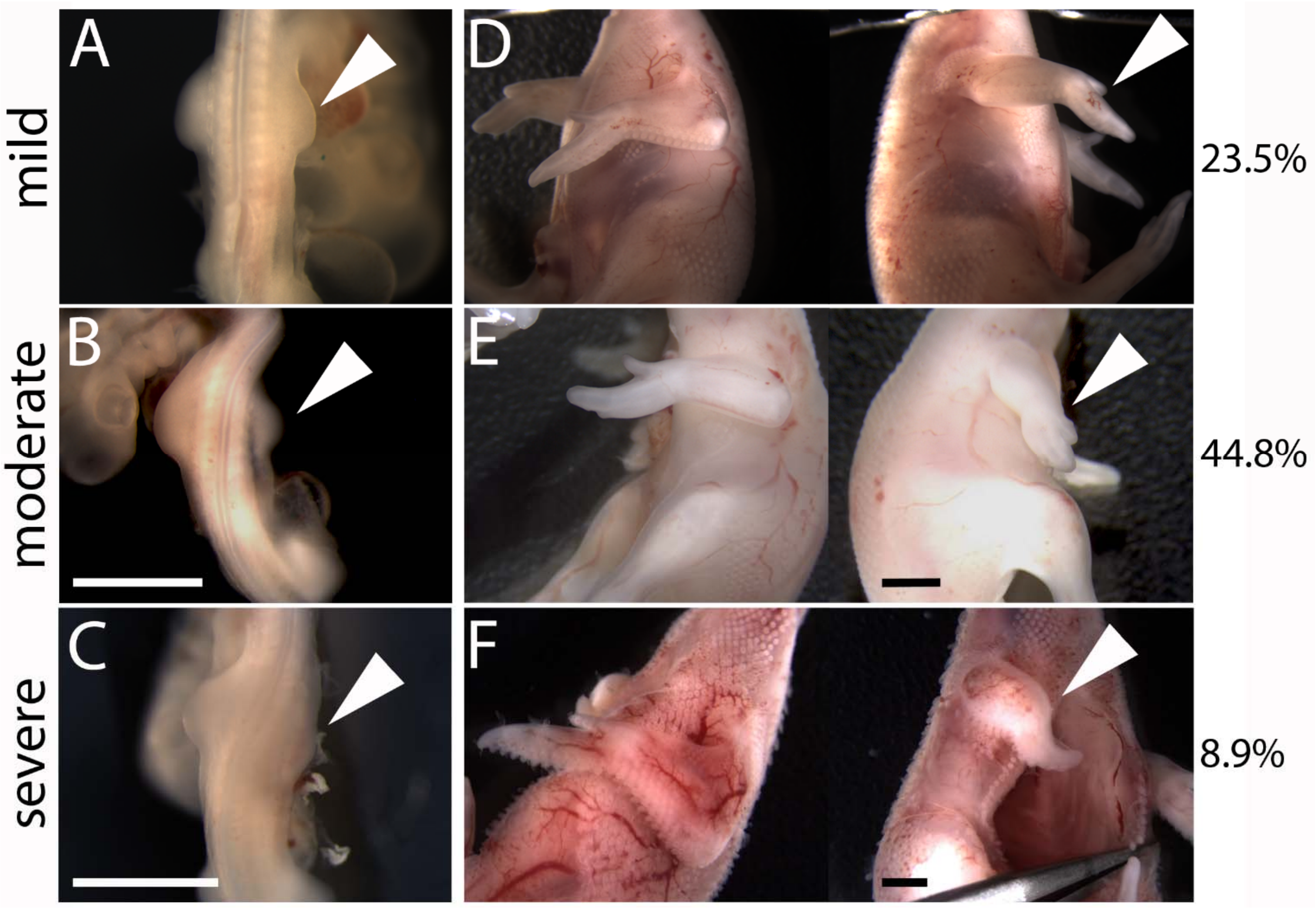

Supplementary Figure 3: The range of phenotypes associated with *Nkx2.5* mis-expression in the chick forelimb. All embryos were infected with *Nkx2.5* expressing virus in the right lateral plate mesoderm at HH10-12 and harvested after (A-C) two days of incubation or (D-F) between 6 and 10 days of incubation. Panels D-F show the uninfected left limb and infected right limb for comparison. The impact of *Nkx2.5* mis-expression was graded as mild, moderate or severe as illustrated. Percentages on the right indicate the proportion of embryos scored in each category out of a total of 281 embryos analysed. No empty vector control infected embryos out of 80 harvested between HH21 (4 days) and 12 days incubation showed wing reductions. Arrowheads indicate affected wing bud/wing. Scale bar = 2 mm.

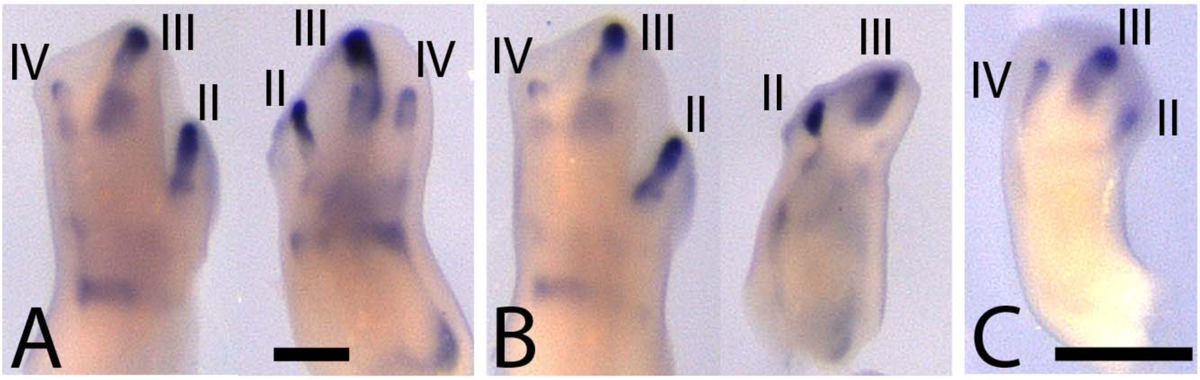

Supplementary Figure 4: Impact of *Nkx2.5* expression on cartilaginous condensations. In situ hybridisation for Sox9 highlighting cartilaginous condensations in (A) a mildly affected chick autopod demonstrating a reduction in digits II and IV, (B) a severely affected chick autopod exhibiting reduced digits II and III and absent digit IV, (C) HH30 emu wing autopod. (A,B) Uninfected wing on left, *Nkx2.5* expressing wing on right. Roman numerals indicate digit numbers. (n = 6 for chick, n = 2 for emu) Scale bar = 1 mm.

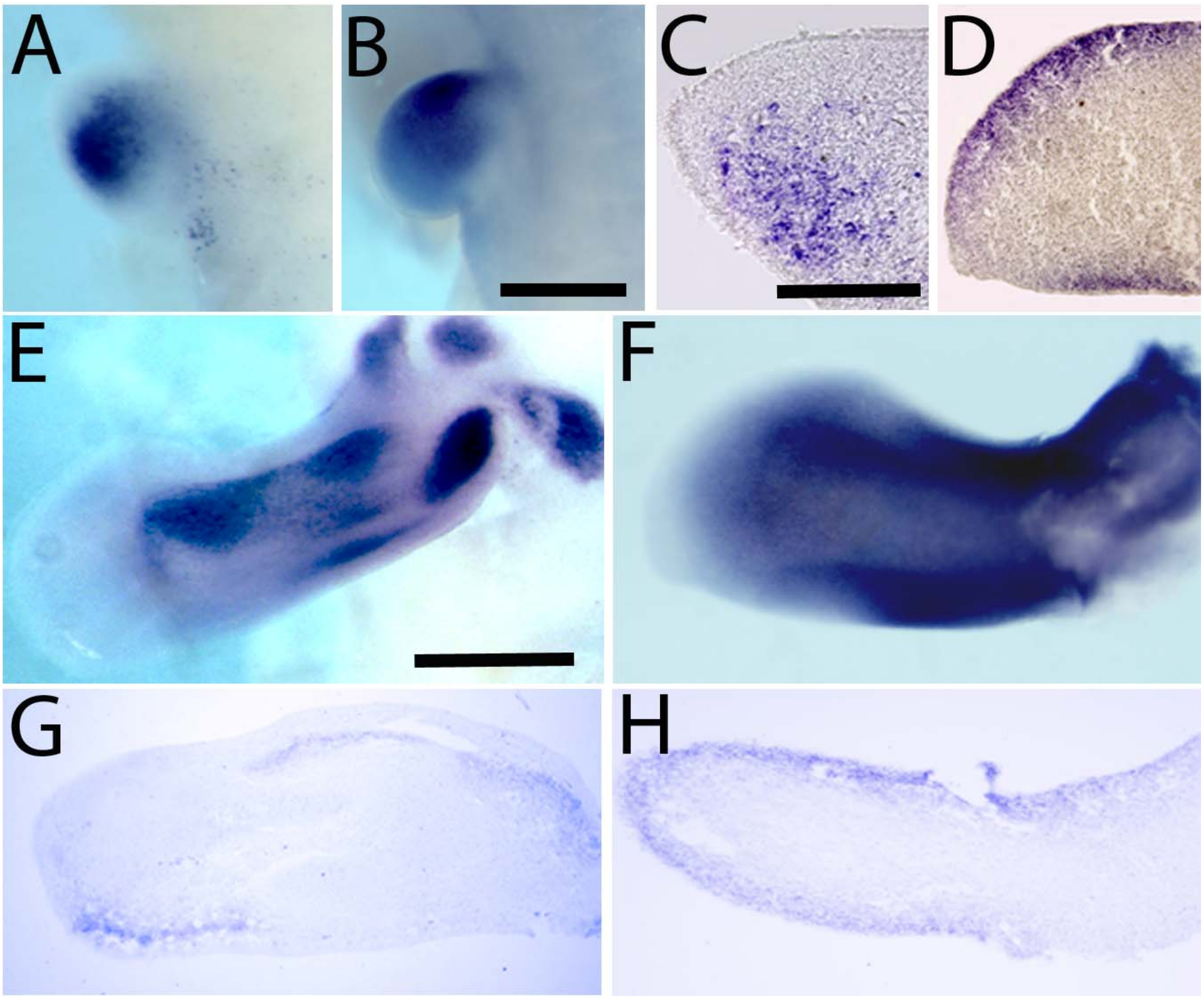

Supplementary Figure 5: Comparison of *Nkx2.5* and *Tcf4* expression domains in embryonic emu limbs. A) *Nkx2.5* mRNA HH25 emu forelimb bud, B) *Tfc4* mRNA in HH25 emu forelimb bud, C) Section of (A), showing restricted expression domain of *Nkx2.5*, D) Section of (B), showing peripheral expression domain of *Tcf4*, E) *Nkx2.5* mRNA HH30 emu forelimb bud, F) *Tfc4* mRNA in HH30 emu forelimb bud, G) Section of HH30 forelimb bud, showing *Nkx2.5* expression, H) Section of HH30 forelimb bud, showing *Tcf4* expression. Images are representative of two or more embryos at each stage. Scale bar (B) = 0.5 mm, (C) = 0.3 mm, (E) = 1mm.

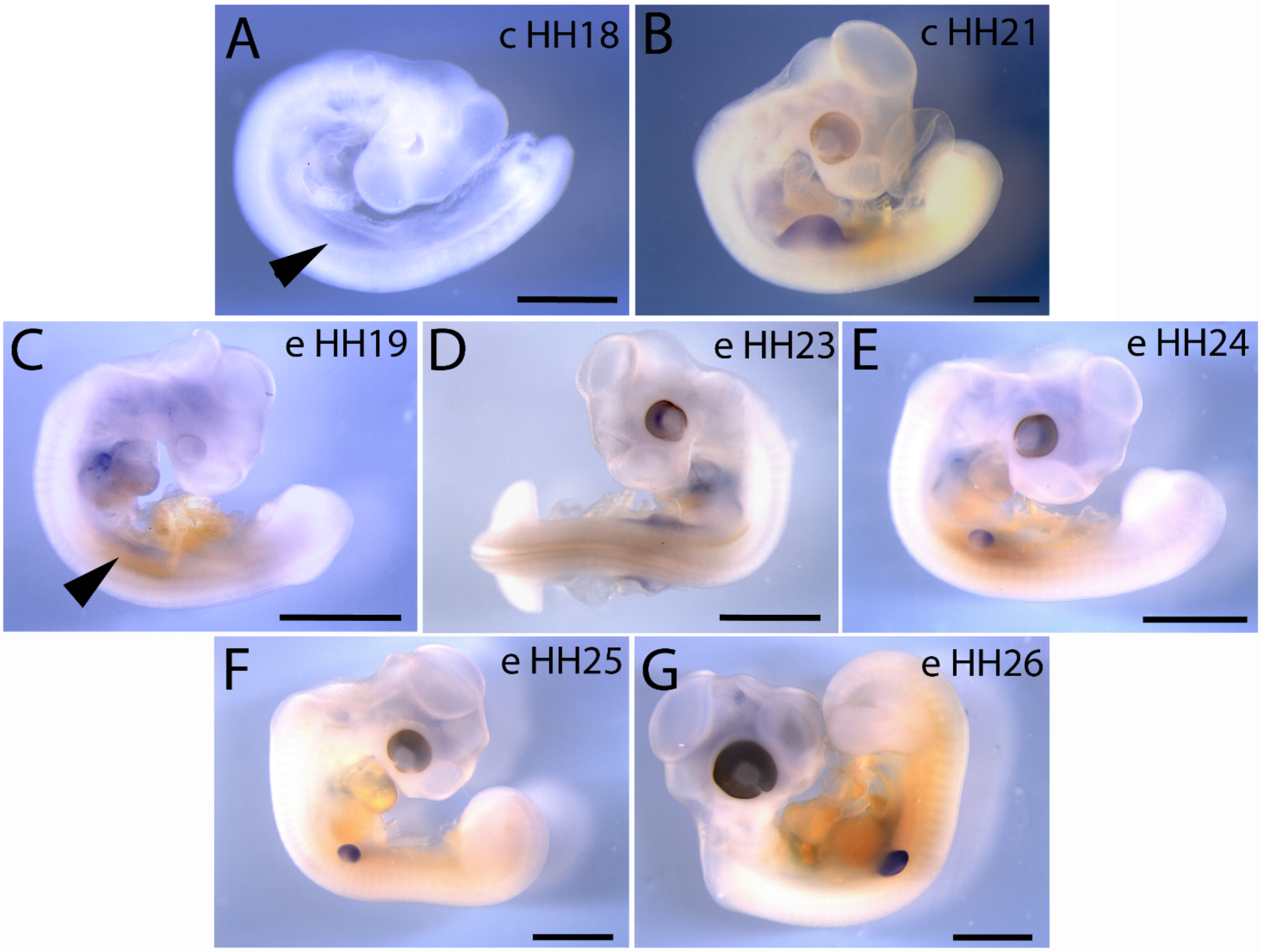

Supplementary Figure 6: Comparison of *Tbx5* expression in chick and emu. (A, B) chick, (C-G) emu. Arrows indicate Tbx5 in the early limb bud. Scale bar = 1 mm (A,B), 2mm (C-G). Images are representative of 2 or 3 embryos at each stage.

Supplementary Table 1: The top 30 most highly ranked genes differentially expressed in a comparison between HH20 chick fore- and hindlimb and emu fore- and hindlimb.

	Gene	Adjusted p-value
ENSGALG00000002886	NKX2-5	0.000171346
ENSGALG00000027513-ENSGALG00000001276	NA	1.46E-05
ENSGALG00000013193	IRX2	0.006557649
ENSGALG00000009192	SLITRK2	1.46E-05
ENSGALG00000015143	TTR	0.016640149
ENSGALG00000027692	KIAA1024L	0.018943315
ENSGALG00000007489	CNNM1	0.001032586
ENSGALG00000020738	NA	0.033439783
ENSGALG00000028158	FGF7	0.000276628
ENSGALG00000015544	ALDOB	0.007763208
ENSGALG00000004804	TGM3	0.035299683
ENSGALG00000000607	GPR37L1	0.009821087
ENSGALG00000008135	SATB2	0.009397886
ENSGALG00000015970	COL9A1	0.002930895
ENSGALG00000007744	CASS4	0.019177523
ENSGALG00000016738	NIPA1	0.026905798
ENSGALG00000006497	ZIC3	0.025548471
ENSGALG00000007508	HPSE2	0.000344306
ENSGALG00000010901	NA	0.032876306
ENSGALG00000025906	HS6ST3	0.046450851
ENSGALG00000004376	IRK1	0.000712562
ENSGALG00000027903	HOXB5	0.002828657
ENSGALG00000006325	NA	0.018715053
ENSGALG00000014699	MEGF10	0.006475108
ENSGALG00000013503	FAM149A	0.002766615
ENSGALG00000025792	C9ORF4	0.006600975
ENSGALG00000007129	ALDH6	0.008858873
ENSGALG00000002769	DNAJC12	0.000632057
ENSGALG00000003952-ENSGALG00000014365	NA	0.006648634
ENSGALG00000013455	HOXB3	0.01340506

For full dataset, see Supplementary Data 1.

Supplementary Table 2. Genes exhibiting statistically significant differential expression between control and *Nkx2.5* mis-expressing chick forelimb buds.

	Symbol	PositionGalGal4	Log2FoldChange	AdjustedPValue
ENSGALG00000002886	NKX2-5	chr13:8535720-8537935	15.03276	0.000279
ENSGALG00000002242	GALNT9	chr15:2342101-2458959	1.492026	0.008334
ENSGALG00000026903	CALB2	chr11:1479309-1484634	7.951976	0.012265
ENSGALG00000002893	STC2	chr13:8561503-8571173	1.642509	0.012265
ENSGALG00000011893	SLC38A6	chr5:54134020-54169085	0.817563	0.012265
ENSGALG00000009693	CPXM2	chr6:31278696-31344582	1.056924	0.012265
ENSGALG00000009393	CAV1	chr1:24999076-25013429	2.352502	0.012265
ENSGALG00000013892	MAP3K5	chr3:54290127-54341428	1.336367	0.012265
ENSGALG00000013085	PTPRO	chr1:62933510-63081609	1.402995	0.016656
ENSGALG00000009280	NPY2R	chr4:20013682-20014840	-1.14005	0.016656
ENSGALG00000027633	RASSF9	chr1:42006974-42038172	0.858071	0.021771
ENSGALG00000010331	MME	chr9:22527164-22563563	0.96464	0.023051
ENSGALG00000007000	NR2F2	chr10:15328162-15333861	-0.94336	0.026814
ENSGALG00000020522	MYRF	chr5:254168-276875	-0.69134	0.028007
ENSGALG00000020292	GABRG4	chr4:10807766-10840783	3.340812	0.037477
ENSGALG00000007113	TNC	chr17:2640784-2707385	1.122254	0.037477
ENSGALG00000016293	BMP5	chr3:86766921-86825286	1.056103	0.037477
ENSGALG00000025822	CYP1B1	chr3:31278222-31281746	1.021709	0.037477
ENSGALG00000005643	LYVE1	chr5:8332203-8342809	1.841351	0.040469
ENSGALG00000009216	NA	chr3:16284705-16286434	-1.82641	0.040469
ENSGALG00000016281	DMD	chr1:114233666-114932830	0.618749	0.041963
ENSGALG00000004596	AR	chr4:429899-473927	-1.28682	0.045707
ENSGALG00000012956	TMTC1	chr1:59846667-59987218	-0.70643	0.046185
ENSGALG00000028946	CSRP2	chr1:38160284-38171384	0.858596	0.046185
ENSGALG00000011271	LUM	chr1:43860882-43872867	-0.92698	0.046696
ENSGALG00000007011	ZBTB16	chr24:4460400-4534848	-1.16698	0.049782

Supplementary Table 3: Key limb patterning genes unresponsive to Nkx2.5 misexpression.

	Symbol	Log2Fold Change	Adjusted p-Value
ENSGALG00000008253	TBX5	0.128704129	0.894156583
ENSGALG00000007706	FGF8	-0.20911757	0.814727413
ENSGALG00000026059	TWIST1	-0.401799008	0.331254278
ENSGALG00000011207	MKP3	-0.015169292	0.972754946
ENSGALG00000009273	HOXD11	0.056078865	0.875203824
ENSGALG00000009274	HOXD12	0.15645927	0.830300409
ENSGALG00000009277	HOXD13	0.388618616	0.440687373
ENSGALG00000006379	SHH	0.468342998	0.304716115
ENSGALG00000012620	PTCH1	0.215264041	0.589396455
ENSGALG00000010133	PTCH2	0.176489867	0.77115247
ENSGALG00000012329	GLI3	-0.173994389	0.598738787
ENSGALG00000008830	BMP2	0.373311633	0.422048793
ENSGALG00000007668	BMP7	0.4194799	0.241535761
ENSGALG00000009724	GREM1	0.007652903	0.995277473
ENSGALG00000026180	MSX2	0.084213225	0.861855126

For full dataset, see Supplementary Data 2.
